# Active surveillance or surgical resection? A survey of treatment strategies for low-risk papillary thyroid microcarcinoma in adults

**DOI:** 10.3389/fonc.2026.1870202

**Published:** 2026-06-18

**Authors:** Xia Qin, Ming-tao Chang, Qiao-zhi Feng, Rui Feng, Jian-fa Chen

**Affiliations:** Department of General Surgery, First Naval Hospital of Southern Command, Zhanjiang, China

**Keywords:** active surveillance, papillary thyroid microcarcinoma, questionnaire survey, surgical resection, treatment strategies

## Abstract

**Objective:**

The purpose of this study was to investigate the clinical status of active monitoring [active surveillance (AS)] or surgical resection for adult low-risk papillary thyroid microcarcinoma (cT1aN0M0 PTMC) in the military and some local medical institutions in southern China.

**Methods:**

A questionnaire survey was conducted on the actual treatment mode of adult PTMC patients in the member institutions of the Southern Theater Command General Surgery Specialty Alliance. The respondents mainly included surgeons engaged in the surgical treatment of thyroid diseases.

**Results:**

A total of 36 medical institutions received replies, and the average annual volume of thyroid surgery in these institutions exceeded 100 cases. For suspicious nodules detected via ultrasound, routine fine-needle aspiration cytology (FNAC) for all suspicious nodules was recommended by six institutions (16.7%); nine (25.0%) performed FNAC only for nodules larger than 10 mm. After diagnosis, AS was recommended by six institutions (16.7%), immediate surgery was recommended by six institutions (16.7%), and 13 institutions (36.1%) left the treatment decision to the patient. For the AS protocol, 22 institutions recommended initial monitoring every 3 months, and 11 recommended every 6 months. When obvious clinical symptoms, new lymph node metastasis, or extrathyroidal invasion appeared, these institutions tended to convert from AS to surgery. During the continuous 3-month period selected by each institution from 2024 to 2025, among 474 PTMC patients, 228 (48.1%) underwent immediate surgery as initial treatment, 175 (36.9%) chose initial AS, and 63 (13.3%) received radiofrequency/microwave ablation. Of the 175 patients initially managed with AS, 49 (28.0% of the AS group) subsequently underwent surgery. Correlation analysis showed that the number of patients undergoing surgery was significantly higher in general surgery departments and in institutions with a larger number of thyroid surgeons (p < 0.05). At the same time, the number of patients receiving AS was significantly higher in tertiary hospitals (p < 0.05).

**Conclusion:**

Within our specialized alliance member institutions, more than one-third of low-risk PTMC patients have adopted AS as their management strategy. However, significant variability exists across medical institutions regarding the indications and implementation protocols for AS. To facilitate the broader and more standardized application of AS, it is essential to strengthen educational efforts directed at both clinicians and patients and to develop more refined, population-specific clinical guidelines or expert consensus for the management of low-risk PTMC in the Chinese population.

## 1 Introduction

In recent years, the incidence of papillary thyroid microcarcinoma (PTMC) has increased significantly, but its mortality rate is still low (1, 2), which has led to a wide discussion on the need for surgical treatment of PTMC. In a retrospective study spanning 30 years, only five of 759 PTMC patients developed clinically significant lymph node metastases, and no patients died due to the disease, suggesting that PTMC is often a biologically indolent tumor (3). Despite this, the management of PTMC remains controversial. Studies have pointed out that while most PTMC cases are indolent, some patients may exhibit more aggressive behavior, especially in the presence of lymph node metastasis or extrathyroidal extension of microcarcinoma (4). Therefore, the necessity of surgical treatment lies in the identification and management of patients with these high-risk features. For patients with low-risk PTMC, studies have shown that overtreatment can be avoided by active surveillance (AS), an approach that was validated in studies at Kuma Hospital and Tokyo Cancer Institute Hospital in Japan (5, 6). AS, as a management strategy, has been shown to be safe and effective in patients with low-risk PTMC. Studies have shown that only 8% of patients who choose AS have tumor enlargement, 3.8% have lymph node metastasis within 10 years, patients who choose immediate surgery experience more adverse events, and the economic cost of AS is significantly lower than that of immediate surgery (7). However, AS still faces challenges in clinical practice in China, and many clinicians are skeptical of this approach, mainly concerning the potential risk of metastasis and patients’ preference for surgery (8). It is still unclear how to manage asymptomatic PTMC. Therefore, this study conducted a questionnaire survey among the member institutions of the General Surgery Specialty Alliance in the Southern Theater Command to understand the current management status of asymptomatic PTMC in the military and some local medical institutions in southern China.

## 2 Methods

### 2.1 Survey overview

On October 18, 2025, a questionnaire titled “Investigation on Current Treatment Strategies for Adult Low Risk Papillary Microcarcinoma (cT1aN0M0 PTMC)” was distributed to 36 medical institutions of the Southern Theater Command General Surgery Specialty Alliance through on-site conference distribution to all surgeons in these institutions who performed thyroid or head and neck surgery, with each institution performing an average of over 100 thyroid surgeries annually. Each medical institution was allowed to submit only one questionnaire response; however, if an institution had two subspecialties or clinical departments that both treated PTMC patients, it was permitted to submit two responses. Responses were required to include the name of the medical institution and the respondent and were collected by a member of the research team. Each reply was expected to represent the basic policy of the institution, not the personal opinion of the respondent. The final questionnaire collection date was October 25, 2025. This study obtained First Naval Hospital of Southern Command ethics committee approval (Number 2026PW002).

### 2.2 Questionnaire

The design and content of the questionnaire were discussed and finalized by members of our research group. The questionnaire mainly included five clinical questions covering the diagnosis and evaluation criteria, treatment strategies and clinical decisions, and specific practices of adult low-risk PTMC. Each question focused on the institution’s clinical experience, and these questions were confined to the management of adult PTMC patients.

Question 1. About fine-needle aspiration cytology (FNAC) indications, please select the primary indication for FNAC in thyroid nodules currently used by your institution:

- Nodule diameter >10 mm.
- Nodule diameter >5 mm with ultrasonic suspicious characteristics (such as low echo, edge blur, vertical growth, and microcalcification).
- Any size, but with highly suspicious ultrasound features (such as breaking through the capsule and confirming lymph node metastasis).
- According to Thyroid Imaging Reporting and Data System (TI-RADS) grading, puncture upon reaching a specific level (such as level 4b or above), regardless of size.
- Other (please note)___________.

Question 2. What is your institution’s preferred initial treatment strategy for low-risk PTMC (cT1aN0M0) diagnosed by cytology?

- Routine recommendation: immediate surgery.
- Introduce two options (surgery/AS) to the patient, but the doctor tends to recommend surgery.
- Objectively introduce two options (surgery/AS) to patients, without expressing a preference.
- Introduce two options (surgery/AS) to patients, but doctors tend to recommend AS as the first choice.
- Other (please briefly explain)___________.

Question 3. Under what circumstances would your institution actively recommend immediate surgical treatment instead of AS? (Multiple choice).

- Multifocal PTMC (tumor count ≥ 2).
- The patient has a family history of differentiated thyroid cancer (first-degree relative).
- Patients with a history of radiation exposure.
- Patient age ≤ 20 years.
- Patient age >60 years.
- The maximum diameter of the tumor is close to 10 mm (e.g., ≥8 mm).
- The tumor is located adjacent to the thyroid capsule or trachea, and ultrasound suspects extrathyroidal invasion (even if not breakthrough).
- Pathology reports suggest high-risk subtypes (such as high cell type and columnar cell type).
- The patient is experiencing severe anxiety due to the presence of a tumor and strongly wishes for surgery.
- The patient has a preconception plan.
- Other (please note)___________.

Question 4. Initial monitoring frequency of AS: □ every 3 months □ every 6 months □ every one year □ other :::_. Until what age should AS be continued? What changes does your institution consider as indications for transitioning from AS to surgery? (Multiple choice).

- Tumor growth: the maximum diameter of the tumor increased by ≥3 mm.
- Lymph node metastasis: new or suspected lymph node metastasis detected via ultrasound.
- Extrathyroidal invasion: ultrasound reveals clear signs of the tumor breaking through the thyroid capsule.
- Clinical progress: obvious clinical symptoms appear (such as hoarseness).
- Patient’s intention: the patient voluntarily requests to terminate AS due to anxiety and other reasons.
- Occurrence of new lesions.

Question 5. Please provide the number of cT1aN0M0 PTMC patients who underwent immediate surgery, AS, AS to surgery, or radiofrequency/microwave ablation treatment at your institution during any continuous 3-month period of your choice (e.g., March–May 2024 or January–March 2025) between January 2024 and September 2025. This approach was adopted to minimize recall bias and improve response feasibility by allowing each institution to select a recent, data-complete quarter.

### 2.3 Statistical analysis

Use SPSS 26.0 statistical software for data processing and statistical analysis. Count the number of data cases (percentage) and use the chi-square test or exact probability method. The measurement data are expressed as mean ± standard deviation (x ± s) and analyzed using an independent-samples t-test or ANOVA. p < 0.05 indicates a statistically significant difference.

## 3 Results

### 3.1 Questionnaire response rate and institutional overview

After repeated follow-up by telephone and email and on−site visits, all 36 medical institutions in the Southern Theater Command General Surgery Specialty Alliance finally responded, yielding a 100% response rate. Among these 36 institutions, 26 (72.2%) were in the general surgery department, six (16.7%) were in the thyroid surgery specialty, and the remaining four (11.1%) were in other departments. The number of thyroid surgeons per department ranged from two to eight. Out of 36 medical institutions, 24 (66.7%) were classified as Grade 3A, seven (19.4%) were classified as Grade 3B, and five (13.9%) were classified as Grade 2A, as shown in **Table 1**.

**Table 1:** Characteristics of responding institutions (N = 36).

Projects	Number of cases (%)
Department	
General surgery	26 (72.2%)
Specialty in thyroid surgery	6 (16.7%)
Other	4 (11.1%)
Number of thyroid surgeons	
2	6 (16.7%)
3–5	18 (50.0%)
6–8	12 (33.3%)
Medical institution level	
Grade 3A	24 (66.7%)
Grade 3B	7 (19.4%)
Grade 2A	5 (13.9%)

### 3.2 Response to questions

The responses to the questionnaire were as follows.

#### 3.2.1 Question 1. Current main indications for FNAC

**Figure 1:**
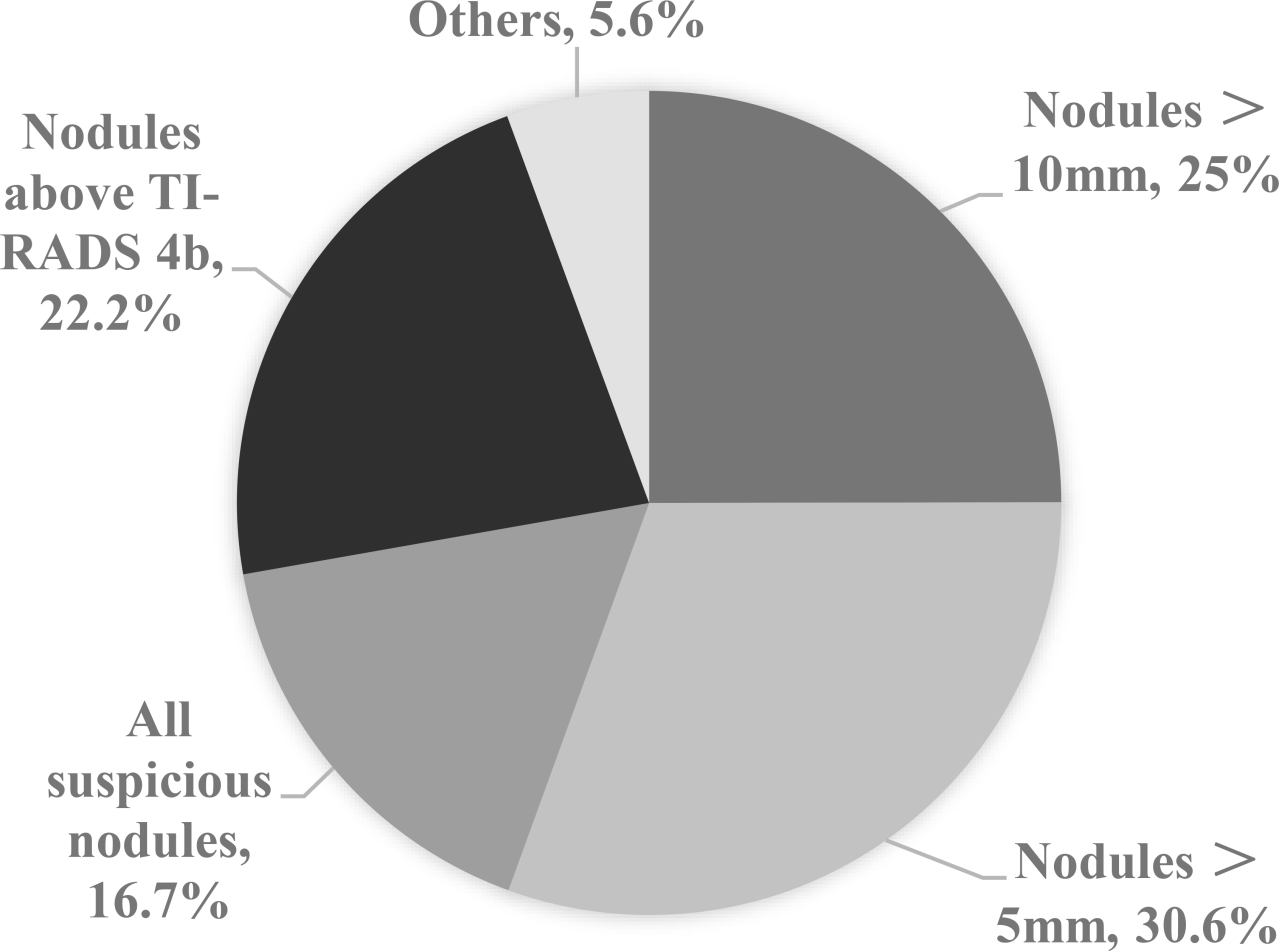
Indications for FNAC of thyroid nodules with suspicious ultrasound features. FNAC, fine-needle aspiration cytology.

As shown in **Figure 1**, regarding the primary indication for FNAC in thyroid nodules with suspicious ultrasound features, 11 institutions (30.6%) performed FNAC on nodules larger than 5 mm with suspicious ultrasound features, nine institutions (25.0%) performed FNAC only for nodules 10 mm, and six (16.7%) of institutions performed FNAC on nodules of all sizes with highly suspicious ultrasound features. Eight institutions (22.2%) considered that nodules reaching a specific TI-RADS level (e.g., 4b or above) should undergo immediate FNAC regardless of size. Two institutions (5.6%) reported other individualized criteria.

#### 3.2.2 Question 2. The current preferred initial treatment strategy for low-risk PTMC diagnosed by cytology

**Figure 2:**
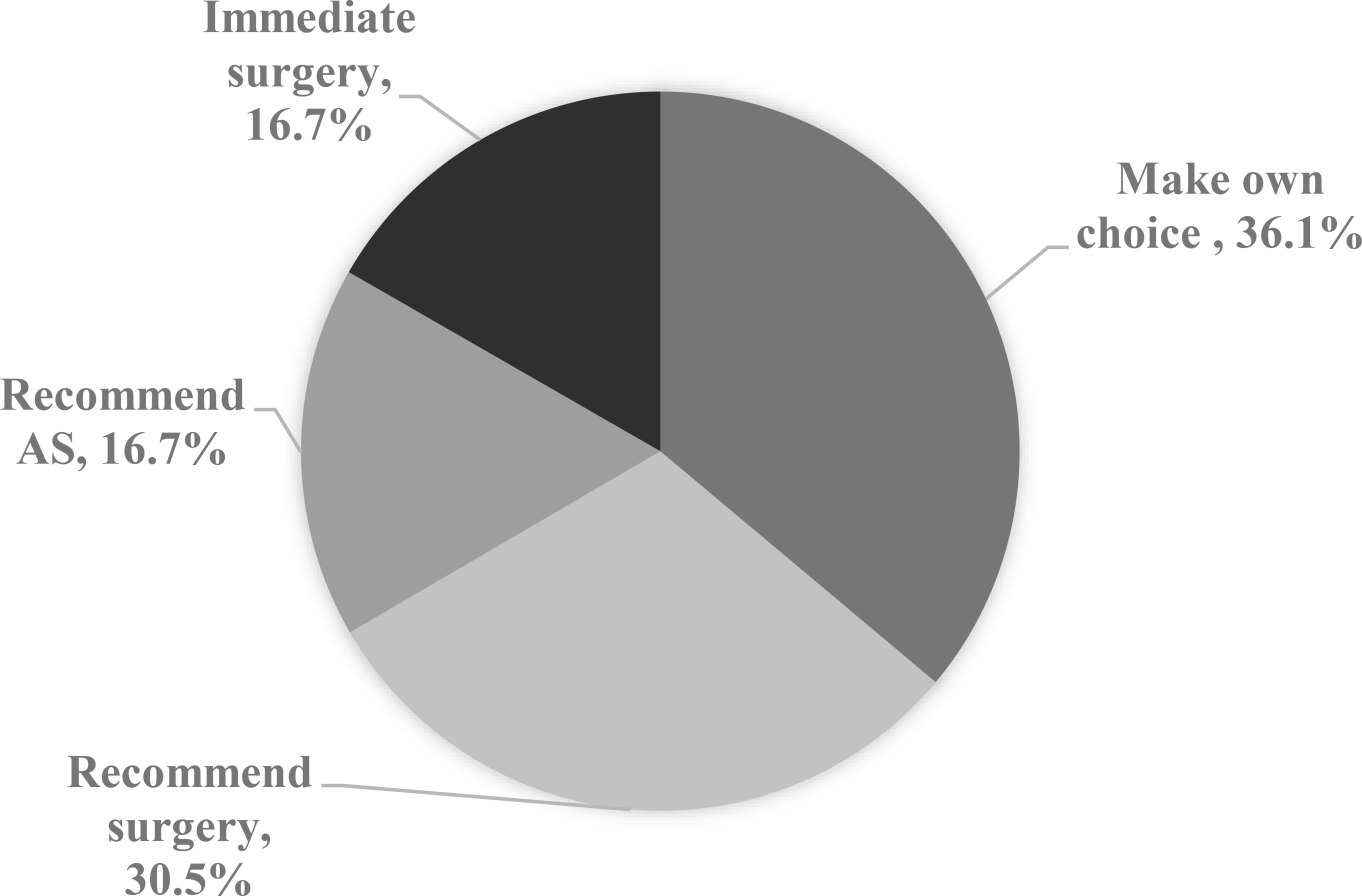
The current treatment strategies for low-risk PTMC. PTMC, papillary thyroid microcarcinoma.

Most responding institutions offer patients two options, namely, AS and surgery. The most common approach (36.1%) was for doctors not to indicate a preference, leaving the decision to patients. Eleven institutions (30.5%) recommended surgery as the first−line treatment, six institutions (16.7%) recommended AS as the first−line treatment, and six institutions (16.7%) routinely recommended immediate surgery (**Figure 2**).

#### 3.2.3 Question 3. Indications for actively recommending surgery for low−risk PTMC

**Figure 3:**
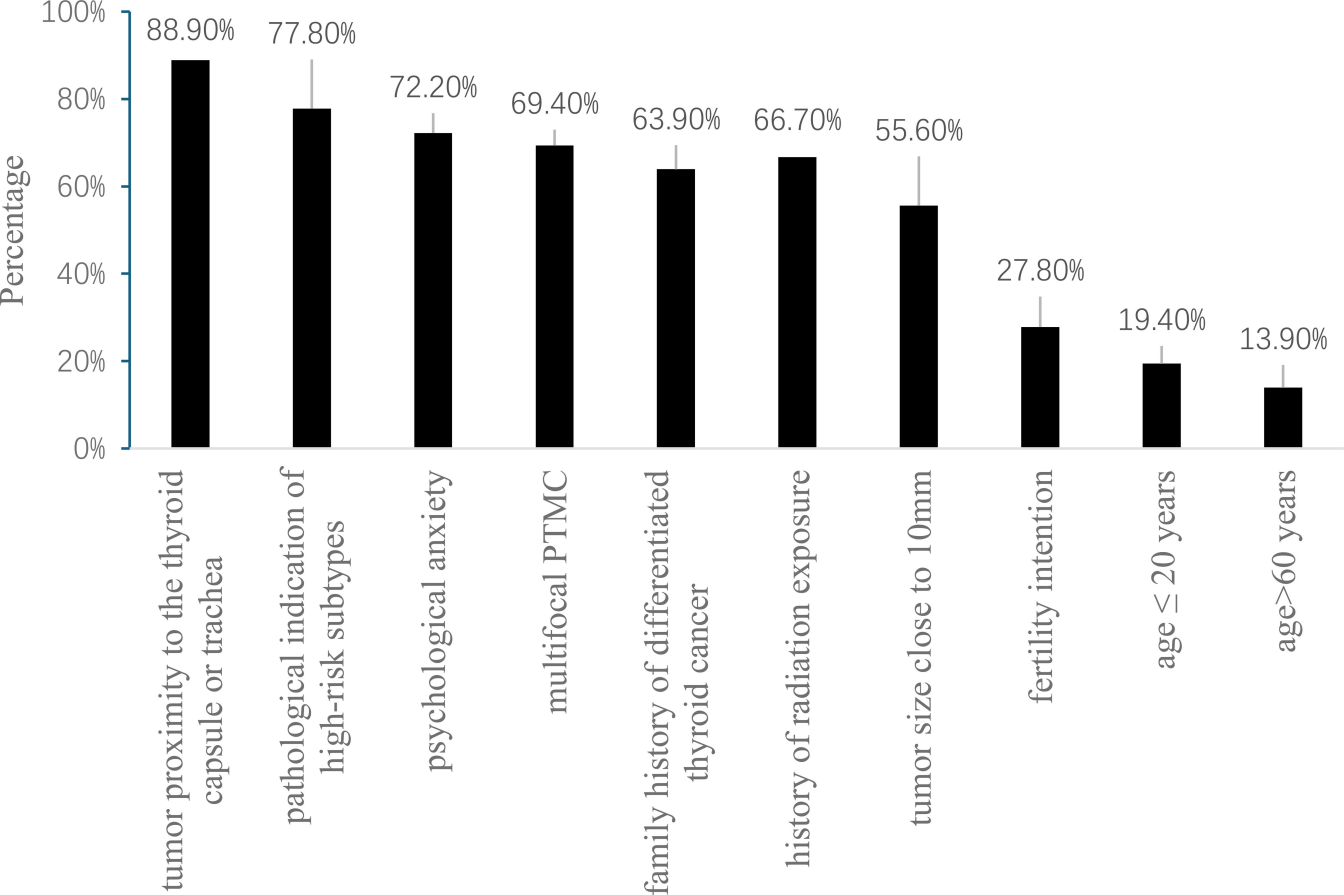
Indications for actively recommending surgery for low-risk PTMC. PTMC, papillary thyroid microcarcinoma.

As shown in **Figure 3**, 36 institutions listed one or more conditions that actively recommend low-risk PTMC surgery, including tumor proximity to the thyroid capsule or trachea (32/36, 88.9%), pathological indication of high-risk subtypes (28/36, 77.8%), psychological anxiety (26/36, 72.2%), multifocal PTMC (25/36, 69.4%), family history of differentiated thyroid cancer (23/36, 63.9%), history of radiation exposure (24/36, 66.7%), tumor size close to 10 mm (20/36, 55.6%), fertility intention (10/36, 27.8%), age ≤ 20 years (7/36, 19.4%), and age > 60 years (5/36, 13.9%).

#### 3.2.4 Question 4. Initial monitoring frequency and conversion triggers for AS

**Figure 4:**
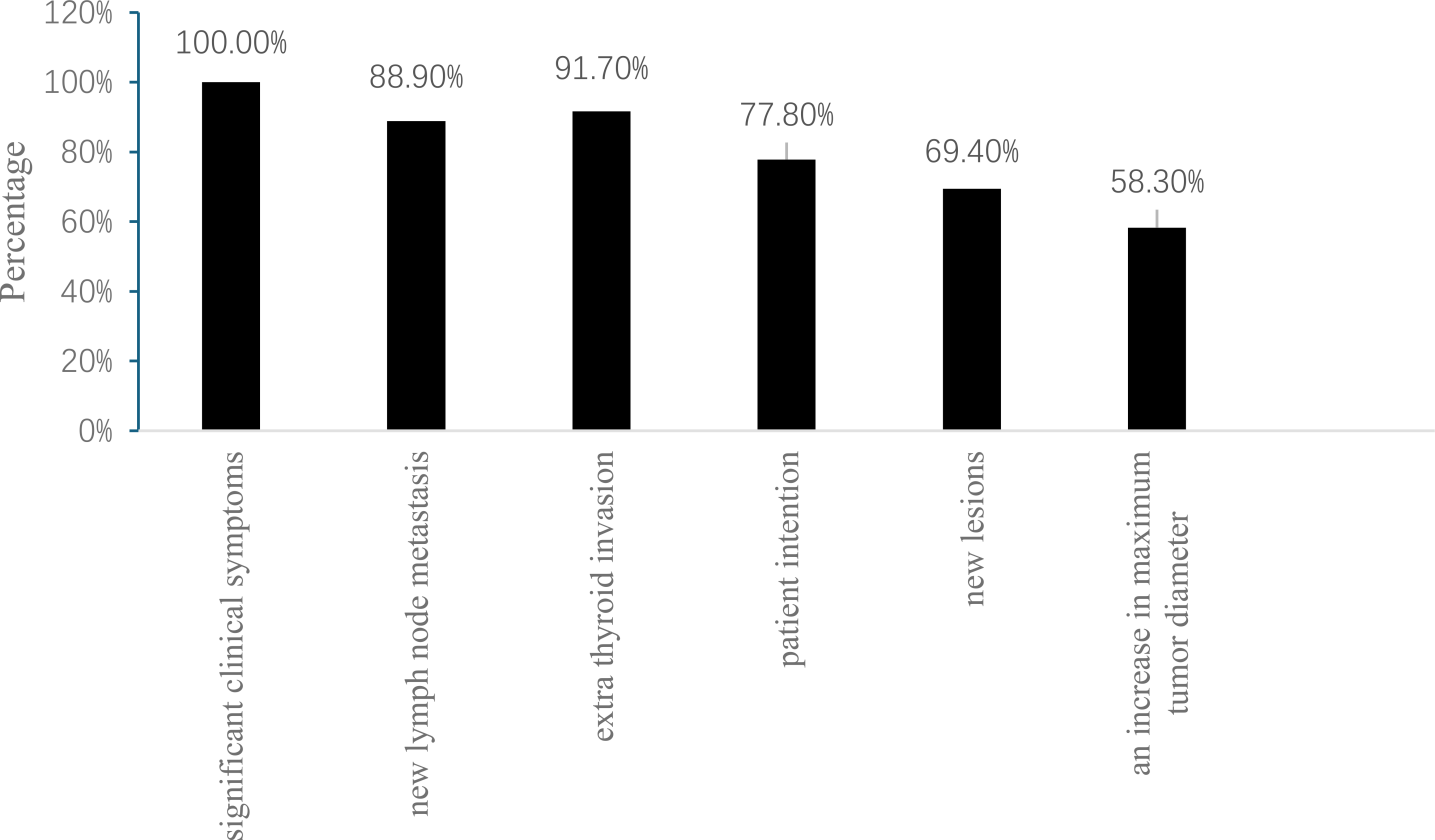
Indications for transitioning from AS to surgery. AS, active surveillance.

Of the 36 institutions, 22 (61.1%) recommended initial monitoring every 3 months, 11 (30.6%) recommended monitoring every 6 months, and three (8.3%) recommended monitoring every 1 year. For the indications for transitioning from AS to surgery, as shown in **Figure 4**, the 36 institutions listed the following triggers: including the appearance of significant clinical symptoms in clinical progression (36/36, 100%), new lymph node metastasis (32/36, 88.9%), extrathyroidal invasion (33/36, 91.7%), patient intention (28/36, 77.8%), the appearance of new lesions (25/36, 69.4%), and an increase in maximum tumor diameter of ≥3 mm (21/36, 58.3%).

#### 3.2.5 Question 5. Actual treatment distribution

**Figure 5:**
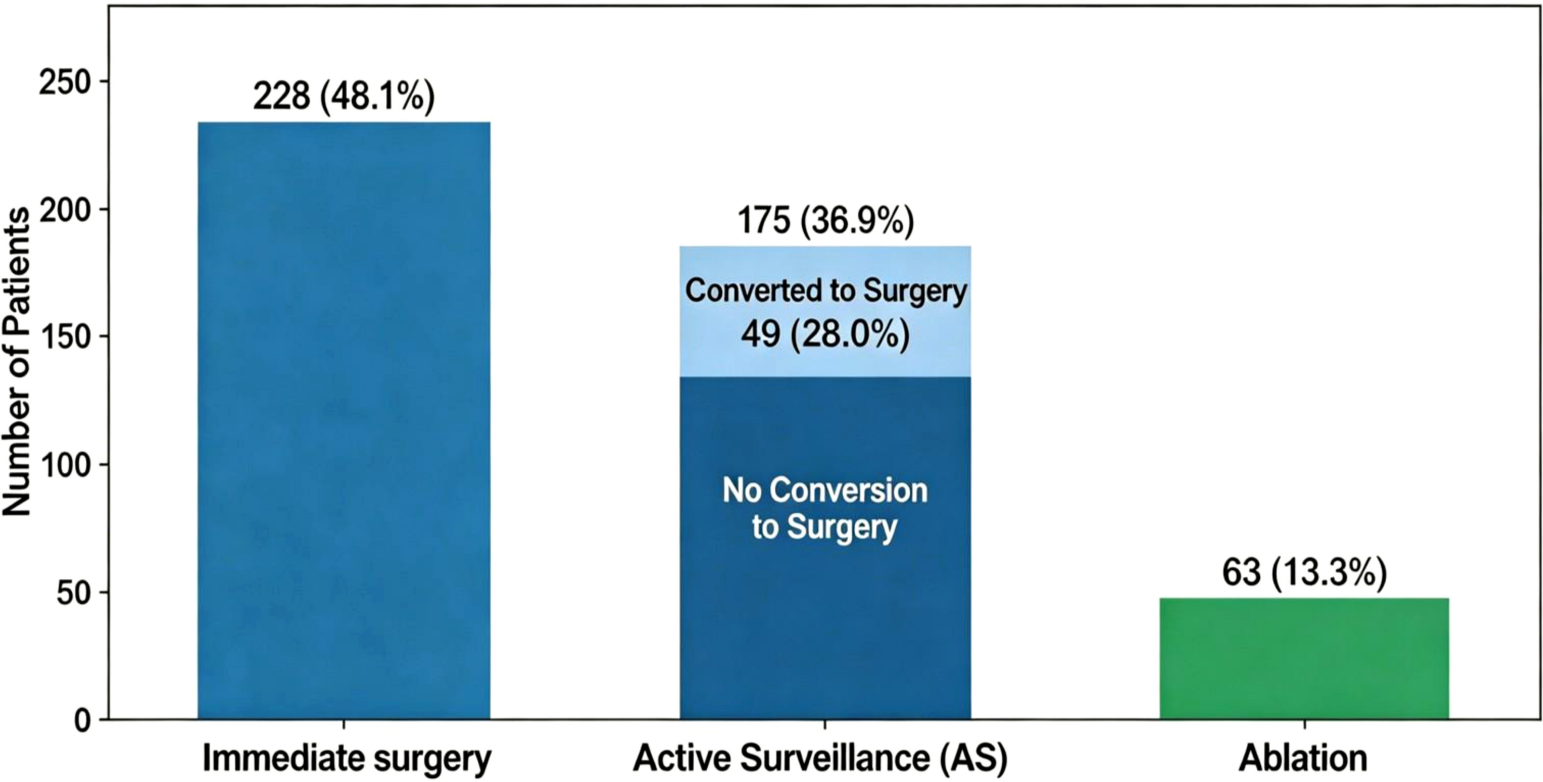
Treatment distribution of 474 PTMC patients from 36 institutions. The bars represent initial treatment allocation (immediate surgery, initial AS, and ablation) and the proportion of AS patients who subsequently converted to surgery. PTMC, papillary thyroid microcarcinoma; AS, active surveillance.

The survey results showed that a total of 474 PTMC patients were treated by 36 institutions (range, 2–65 cases; mean, 13.2 cases). Of these, 228 patients (48.1%) underwent immediate surgery as their initial treatment, 175 patients (36.9%) chose AS as their initial management, and 63 patients (13.3%) received radiofrequency/microwave ablation as a primary treatment. Among the 175 patients initially managed with AS, 49 (28.0% of the AS group and 10.3% of the total cohort) subsequently switched to surgery during the observation period (**Figure 5**).

### 3.3 Correlation analysis between low-risk PTMC management strategies and characteristics of medical institutions

As shown in **Table 2**, the indications for FNAC are independent of various characteristics of medical institutions, including department type, the number of thyroid surgeons, and the level of medical institution (all p > 0.05), indicating that this decision relies more on individualized clinical judgment rather than differences in institutional resources.

**Table 2:** Relationship between FNAC and the characteristics of medical institutions (N = 36).

Projects	Indications of FNAC			χ^2^	p
	All suspicious nodules ^†^	>5-mm nodules	>10-mm nodules		
Department				0.379	0.984
General surgery	11 (42.3%)	8 (30.8%)	7 (26.9%)		
Thyroid specialty	3 (50.0%)	2 (33.3%)	1 (16.7%)		
Others	2 (50.0%)	1 (25.0%)	1 (25.0%)		
Number of thyroid surgeons				0.681	0.954
2	3 (50.0%)	2 (33.3%)	1 (16.7%)		
3–5	7 (38.9%)	6 (33.3%)	5 (27.8%)		
6–8	6 (50.0%)	3 (25.0%)	3 (25.0%)		
Medical institution level				1.329	0.856
Grade 3A	11 (45.8%)	7 (29.2%)	6 (25.0%)		
Grade 3B	2 (28.6%)	3 (42.9%)	2 (28.6%)		
Grade 2A	3 (60.0%)	1 (20.0%)	1 (20.0%)		

FNAC, fine-needle aspiration cytology.

^†^ “All suspicious nodules” includes institutions that performed FNAC on all suspicious nodules regardless of size (n =6), those that used TI−RADS grading (≥4b) irrespective of size (n =8), and those that reported other individualized criteria (n =2).

The preferred treatment strategy and management approach are also independent of the department type and number of doctors in the institutional characteristics (**Table 3**). However, the proportion of patients in Grade 3A hospitals who are recommended to use AS as a first-line treatment strategy (20.8%) is higher than that in Grade 3B (14.3%) and 2A hospitals (0%), but the difference has not yet reached a significant level (p = 0.140).

**Table 3:** Relationship between preferred treatment strategies for low-risk PTMC and characteristics of medical institutions (N = 36).

Projects	Treatment strategies			χ^2^	p
	Recommended surgery	Make own decisions	Recommend the AS		
Department				1.904	0.753
General surgery	13 (50.0%)	10 (38.5%)	3 (11.5%)		
Thyroid specialty	2 (33.3%)	2 (33.3%)	2 (33.3%)		
Others	2 (50.0%)	1 (25.0%)	1 (25.0%)		
Number of thyroid surgeons				3.584	0.465
2	4 (66.7%)	2 (33.3%)	0		
3–5	9 (50.0%)	6 (33.3%)	3 (16.7%)		
6–8	4 (33.3%)	5 (41.7%)	3 (25.0%)		
Medical institution level				6.914	0.140
Grade 3A	8 (33.3%)	11(45.8%)	5 (20.8%)		
Grade 3B	5 (71.4%)	1 (14.3%)	1 (14.3%)		
Grade 2A	4 (80.0%)	1 (20.0%)	0		

PTMC, papillary thyroid microcarcinoma; AS, active surveillance.

The relationship between the actual number of patients treated for low-risk PTMC and the characteristics of medical institutions is presented in **Table 4**. Medical institutions with a higher proportion of general surgery departments (56.0%) and a larger number of thyroid surgeons (70.3%) had significantly more patients undergoing surgery (p < 0.05). Additionally, Grade 3A hospitals had significantly more patients undergoing AS (41.6%) (p = 0.005).

**Table 4:** The relationship between the actual number of patients treated with low-risk PTMC and the characteristics of medical institutions.

Projects	The total number	Receiving surgery			Receiving AS		
		Number of patients (%)	χ^2^	P	Number of patients (%)	χ^2^	P
Department			20.779	0.001		3.607	0.165
General surgery	302	169 (56.0%)			121 (40.1%)		
Thyroid specialty	95	34 (35.8%)			29 (30.5%)		
Others	77	25 (32.5%)			25 (32.5%)		
Number of thyroid			47.379	0.001		1.798	0.407
surgeons							
2	98	31 (31.6%)			37 (37.8%)		
3–5	221	88 (39.8%)			75 (33.9%)		
6–8	155	109 (70.3%)			63 (40.6%)		
Medical institution level			0.040	0.980		10.81	0.005
Grade 3A	315	152 (48.3%)			131 (41.6%)		
Grade 3B	93	45 (48.4%)			30 (32.3%)		
Grade 2A	66	31 (47.0%)			14 (21.2%)		

PTMC, papillary thyroid microcarcinoma; AS, active surveillance.

### 3.4 The institutions’ suggestions and recommendations regarding AS

In addition to responding to the five questions in the questionnaire, these institutions provided constructive opinions on AS (**Table 5**), covering four aspects: patient screening and risk assessment for AS, AS implementation and monitoring plans, doctor–patient communication and decision-making assistance, and future research directions.

**Table 5:** Response to the institution's suggestions and recommendations regarding AS.

Advice	Number of institutions mentioned
A. Patient screening and risk assessment	
1. For patients with ≤2 small cancerous lesions and no lymph node metastasis, AS can be cautiously included	9
2. If there are ≥3 lesions or a family history of thyroid cancer in first-degree relatives, it is recommended to strengthen baseline assessment (such as ultrasound + elastography) and shorten the first follow-up interval	3
3. For patients with sustained elevation of serum thyroglobulin (Tg) (>2 times the upper limit of normal) and Thyroid-Stimulating Hormone (TSH) suppression, it is recommended to perform diagnostic ultrasound and molecular testing if necessary to rule out occult progression	7
4. It is recommended to introduce a standardized ultrasound scoring system (such as based on tumor edge, location, internal echo, and calcification type), with a total score ≥ a specific threshold (such as 4 points) as a relative contraindication for AS	18
B. AS implementation and monitoring plan	
1. For patients under 40 years old, AS should be fully informed to persist for a long time (such as more than 10 years)	15
2. Perform high-resolution neck ultrasound (including central and lateral cervical lymph nodes) every 6 months within the first 2 years. After 2 years of stability, it can be extended to once a year	8
3. Routine chest CT or whole-body imaging is not recommended unless ultrasound suggests suspicious metastasis	9
4. Remote/community collaborative follow-up: for patients with good compliance and stable condition, partial follow-up can be conducted through certified community hospitals or remote medical care to reduce the dropout rate	7
5. Institutions should establish an AS-specific disease database to record the AS conversion rate, tumor progression rate, loss to follow-up rate, and patient satisfaction as core indicators for AS quality improvement	4
C. Doctor–patient communication and decision-making assistance	
1. Recommend using visual decision support tools (such as risk–benefit comparison charts and simulated case videos) to help patients understand the long-term differences between AS and surgery and record their decision preferences	3
2. In popular science materials, it should be emphasized that AS does not mean “no treatment”, “all minor cancers are harmless”, or “nodules below 10 mm do not require examination” to avoid misunderstandings	27
3. It is recommended to collect the patient's anxiety self-assessment scale and quality of life score at each follow-up as one of the reference criteria for whether to terminate AS	7
D. Future research directions and data foundation	
1. Establish a nationwide AS registration system and conduct long-term follow-up on PTMC prospective cohort	13
2. Conduct a multicenter retrospective study on a small number of PTMCs with progression or metastasis to clarify their clinical, ultrasound, and molecular characteristics	16
3. Conduct randomized controlled or real-world studies to evaluate whether moderate TSH suppression (such as 0.5–1.0 mIU/L) can reduce tumor growth rate in AS patients	4
4. Compare the tumor control rate, complications, and quality of life of AS and thermal ablation (radiofrequency/microwave) in the same low-risk PTMC, providing evidence for a third pathway for those who are unwilling to undergo surgery or long-term monitoring	21

PTMC, papillary thyroid microcarcinoma; AS, active surveillance.

## 4 Discussion

This study systematically investigated the diagnosis and treatment status of low-risk PTMC in adults for the first time among member units of the General Surgery Specialty Alliance in the Southern Theater Command of China. After repeated follow-up, a 100% response rate was achieved among the 36 member institutions. The high proportion of Grade 3A hospitals (66.7%) suggests that the survey results adequately reflect the practice patterns of major medical centers in the region. This study found that the indications for FNAC exhibit significant practical diversity. Only 25.0% of the institutions followed the traditional threshold of diameter > 10 mm (9), whereas a larger proportion (30.6%) adopted a strategy of >5 mm combined with suspicious ultrasound features, and 16.7% relied solely on TI−RADS grading. This “risk stratification”−oriented FNAC strategy reflects clinicians’ dual consideration of the risks of overdiagnosis and missed diagnosis, in contrast to the conservative recommendations of the 2015 American Thyroid Association (ATA) guidelines (10), and is closer to the positive stance of the Japan Association of Breast and Thyroid Sonology (11). It is worth noting that FNAC indications were not significantly correlated with institutional level, department type, and the number of doctors (p > 0.05), indicating that this decision relies more on individualized clinical judgment rather than differences in institutional resources.

At the treatment strategy level, although most institutions (83.3%) offered AS as an option to patients, the actual treatment distribution shows that nearly half (48.1%) of patients receive immediate surgery, while AS only accounts for 36.9%. This “knowledge–action gap” deserves attention. Further analysis showed that department type and the number of surgeons significantly influenced the surgery rate: the proportion of surgeries in general surgery (56.0%) and institutions with more doctors (70.3%) is significantly higher, which may reflect the “surgical inertia” of surgical leading departments and the tendency of institutions with sufficient manpower to actively intervene. Encouragingly, the AS acceptance rate was significantly higher in Grade 3A hospitals (41.6%) than in Grade 2A hospitals (21.2%, p = 0.005), indicating the positive leading role of high−volume thyroid centers in promoting AS. Research has shown that the practical application of AS worldwide is still influenced by multiple factors. First, patients’ mental health and quality of life are important considerations. Compared with immediate surgery, patients who choose AS perform better in terms of mental health (12). Second, the implementation of AS is also influenced by the healthcare system and cultural background. In countries and regions where diagnostic and treatment strategies are relatively conservative, the application of AS is still limited (13, 14).

Our study revealed several locally characteristic phenomena regarding the indications for actively recommending surgery. Tumors located adjacent to the thyroid capsule or trachea (88.9%) ranked first, reflecting a cautious attitude towards the risk of local invasion. Previous studies have shown that the angle formed between the tumor surface and the tracheal cartilage and the presence of normal edges between the tumor and the capsule are of great significance for evaluating whether the tumor requires surgery (15). Psychological anxiety (72.2%) ranked third, far exceeding the figures reported in Japan (16), indicating that both doctors and patients in China have a heavy psychological burden on cancer diagnosis, and it is particularly urgent to strengthen psychological support and popular science education. In addition, among the indications for AS conversion surgery, “clinical symptoms appear” is 100% recognized, while “tumor growth ≥3 mm” is only 58.3% recognized, suggesting that Chinese physicians hold a relatively conservative attitude toward simple tumor size progression and pay more attention to functional or structural high-risk signs. This is similar to the findings of multiple foreign studies, which suggest that the multiplicity and size of tumors should not be considered contraindications for AS (17–19).

The constructive suggestions from the responding institutions provide important directions for future guideline development. A substantial number of institutions called for the introduction of standardized ultrasound scoring systems to quantify contraindications for AS; many emphasized the need to avoid the public misconception that “AS is equivalent to no treatment”, and several suggested comparative studies between AS and thermal ablation. These suggestions all point to the same core issue: the successful implementation of AS requires not only clinical evidence but also standardized operating systems, precise doctor–patient communication, and diverse treatment options (20, 21).

This study has several limitations. The sample was drawn mainly from tertiary hospitals and may not represent the majority of primary healthcare institutions, potentially overestimating AS acceptance. The investigation was based on institutional policies rather than individual physician decisions, which may have introduced reporting bias. The practice patterns observed in the Southern Theater Command may not be generalizable to the whole country. In the future, it is necessary to conduct a larger-scale investigation and research, including grassroots medical institutions.

In summary, many medical centers in southern China have incorporated AS as a management option for low−risk PTMC; however, its wider adoption still faces obstacles including surgical inertia, patient anxiety, and lack of standardization. To promote the rational application of AS, it will be necessary to develop low−risk PTMC guidelines tailored to the Chinese context, strengthen ultrasound training, create decision support tools, and conduct head−to−head comparative studies between AS and ablation.

## Data Availability

The raw data supporting the conclusions of this article will be made available by the authors, without undue reservation.
